# Searching for Blockers of Dengue and West Nile Virus Viroporins

**DOI:** 10.3390/v14081750

**Published:** 2022-08-11

**Authors:** Hiya Lahiri, Isaiah T. Arkin

**Affiliations:** Department of Biological Chemistry, The Alexander Silberman Institute of Life Sciences, The Hebrew University of Jerusalem, Edmond J. Safra Campus, Jerusalem 9190400, Israel

**Keywords:** ion channel, drug repurposing, antiviral drug, channel blocker

## Abstract

Flavivirus infections, such as those caused by dengue and West Nile viruses, emerge as new challenges for the global healthcare sector. It has been found that these two viruses encode ion channels collectively termed viroporins. Therefore, drug molecules that block such ion-channel activity can serve as potential antiviral agents and may play a primary role in therapeutic purposes. We screened 2839 FDA-approved drugs and compounds in advanced experimental phases using three bacteria-based channel assays to identify such ion channel blockers. We primarily followed a negative genetic screen in which the channel is harmful to the bacteria due to excessive membrane permeabilization that can be relieved by a blocker. Subsequently, we cross-checked the outcome with a positive genetic screen and a pH-dependent assay. The following drugs exhibited potential blocker activities: plerixafor, streptomycin, tranexamic acid, CI-1040, glecaprevir, kasugamycin, and mesna were effective against dengue virus DP1. In contrast, idasanutlin, benzbromarone, 5-azacytidine, and plerixafor were effective against West Nile Virus MgM. These drugs can serve as future antiviral therapeutic agents following subsequent in vitro and in vivo efficacy studies.

## 1. Introduction

The genus Flaviviruses belongs to the Flaviviridae family of enveloped positive-strand RNA viruses. It includes several important arboviral pathogens that represent a specific health burden, such as the dengue virus, West Nile virus, Zika virus, yellow fever virus, and tick-borne encephalitis, to name a few. Dengue fever, as an example, is transmitted by *Aedes* mosquitos and exacts an appreciable toll with ca. 40,000 annual deaths stemming from 390 million infections and half a million hospitalizations [1]. Despite the risk of infection existing in 129 countries, 70% of the actual burden is in Asia. According to the World Health Organization (WHO), dengue cases has been increased 8-fold over the past two decades [2].

West Nile fever, transmitted by Culex mosquitos, is another cause of concern. Identified in Uganda in 1937, it is now spread globally [3]. Since its discovery in 1999, there have been 52,532 cases in the US, of which 25,849 have been neuroinvasive, leading to 2456 deaths [4]. Consequently, West Nile fever is the leading cause of mosquito-borne disease in the continental US.

Prevention options for flavivirus infection focus on transmission containment. At the same time, vaccines are available against some members of the genus, such as yellow fever, Japanese encephalitis, tick-borne encephalitis, and dengue fever. However, the last vaccine is only recommended for individuals who have previously contracted dengue fever since it may increase the probability of a severe outcome for those who have never contracted the disease [5]. Finally, no antiviral drugs are currently approved against flaviviruses [6], reducing treatment options to supportive care.

The non-segmented flavivirus genome is 10–11 kbp in length and codes for a single large protein proteolytically cleaved to approximately a dozen structural and non-structural proteins [3,7]. Amongst these proteins, one can identify the C terminal domain of the M protein of dengue virus and West Nile viruses, which acts as a viroporin. The M protein for dengue virus has been named DP1 (NP_722459.2); for WNV, it is MgM (YP_001527877.1). The length of the two proteins is 75 amino acids, and both are predicted to traverse the lipid bilayer once [8]. As a family, viroporins are found in numerous viruses [9]. Since ion channels as a family are excellent drug targets, it follows that blocking viroporins represents an attractive approach to curb infectivity [10].

One such example is the anti-flu aminoadamantane drugs [11] that target influenza’s M2 protein [12] by blocking its $H^{+}$ channel activity [13]. Widespread viral resistance has rendered both drugs ineffective [14], emphasizing the importance of continuous drug development and effective resistance mapping [15].

Considering the paucity of antiviral agents against flaviviruses, we set forth to identify blockers of viroporins from two flaviviruses: dengue virus and West Nile virus. We employed three bacteria-based assays to characterize the protein’s channel activity and then screened a drug repurposing library against each target. Our results have yielded four blockers against each channel that may represent a starting point for an antiviral drug discovery program.

## 2. Materials and Methods

### 2.1. Bacteria-Based Channel Assays

Three bacteria-based channel assays were employed to investigate the viroporin channel activity and blockers thereof. In all assays, the MBP (maltose-binding protein) fusion purification system (New England BioLabs; Ipswich, MA, USA) was used, where dengue virus DP1 and West Nile virus MgM were expressed as a chimera by fusion to the carboxy-terminal of the maltose-binding protein. Previous results have shown that utilizing this construct results in functional expression in bacteria [8,15,16,17,18,19,20].

#### 2.1.1. Negative Assay

Bacteria cultures (DH10B) were grown overnight and finally diluted 500-fold and again set to grow until their O.D._600_ reached 0.2. From the culture, 50 μL were added to 96-well flat-bottomed plates, pre-treated with 50 μL of required staff. Induction was achieved by employing different concentrations of isopropyl-β-d-1-thiogalactopyranoside. A multi-plate incubator (infinite M200 pro, Tecan Group; Männedorf, Switzerland or LogPhase 600 from BioTek; Santa Clara, CA, USA) was used to incubate the plates for 16 h at 37 °C at a constant shaking rate (700 rpm). Bacterial growth was monitored by measuring O.D._600_ every 15 min. Duplicates or triplicates were conducted for every measurement.

#### 2.1.2. Positive Assay

The positive assay employed the same protocol as the negative assay, but a $K^{+}$-uptake deficient bacteria strain was used [21]. Additionally, overnight growth was conducted in LB media in which 100 mM KCl replaced NaCl.

#### 2.1.3. Acidity Assay

The acidity assay is based on bacteria expressing a chromosomal copy of a pH-sensitive GFP [22]. Overnight bacterial cultures were diluted to 1:500 in LB media and subsequently grown to an O.D._600_ of 0.6–0.8. Protein expression was induced by isopropyl-β-d-1-thiogalactopyranoside at different concentrations, as noted. After one hour of induction, the cells were diluted to an O.D._600_ of 0.2 and pelleted at 3500 g for 10 min. Subsequently, the cells were resuspended in McILvaine Buffer [23], which contains 200 mM Na_2_HPO_4_ and 0.9% NaCl adjusted to pH 7.6 with 0.1 M citric acid. Then, 200 μL of cell suspension were added to a 96-well plate (Nunclon f96 Microwell Black Polystyrene, Thermo Fisher Scientific; Waltham, MA, USA), each well containing 30 μL of McILvaine Buffer. The plate included three wells with McILvaine buffer and three with culture without induction as control. The fluorescent measurements were carried out at an ambient temperature in a microplate reader (Infinite F200 Pro, Tecan Group; Männedorf, Switzerland) with two pairs of bandpass filters: 520 nm emission filter and 390 and 466 nm excitation filters. At the starting point, 70 μL of 300 mM citric acid was added to the bacterial culture, and a fluorescent read-out was taken for 30 s for each wavelength. Finally, proton concentration was calculated from the ratio of two wavelengths [22].

### 2.2. Chemical Screening

A chemical library of 2839 compounds was purchased from MedChem Express (HY-L035, Monmouth Junction, NJ, USA). All chemicals were screened at 100 μM using the negative screening at first, whereby 100 μM isopropyl-β-d-1-thiogalactopyranoside was used to induce DP1 and MgM expression. Bacteria without isopropyl-β-d-1-thiogalactopyranoside (i.e., no protein induction) were used as a positive control, while bacteria that received only DMSO served as the negative control. Drugs that increased bacterial growth to a certain threshold were selected for further duplicate analyses. Subsequently, drugs that passed duplicate analysis in the negative assays were examined by positive assay, where 5 μM and 10 μM isopropyl-β-d-1-thiogalactopyranoside were used to induce DP1 and MgM expression, respectively. Compounds that passed the negative and positive assays were subjected to dose-response analyses and fluorescence-based assays. Finally, the negative assay tested the additive effects of drugs by employing equal concentrations of all possible combinations.

### 2.3. K${}_{i}$ Calculation

The inhibitory constant calculation was conducted for the negative assay results using Prism 9 (GraphPad Software; San Diego, CA, USA). The data were normalized by setting uninduced and induced bacteria growth rates to 1 and 0, respectively. Subsequently, the data are non-linearly fit to the following equation: $a=\left(a_{max}\left[\mathrm{drug}\right]\right)/\left(K_{i}+\left[\mathrm{drug}\right]\right)$, whereby *a* is the normalized activity and $a_{max}$ is the maximal activity. The *K*${}_{i}$ and fit quality (R^2^) are listed in the figure.

### 2.4. Chemicals

The isopropyl-β-d-1-thiogalactopyranoside was purchased from Biochemika-Fluka (Buchs, Switzerland). All other chemicals were purchased from Sigma-Aldrich laboratories (Rehovot, Israel).

### 2.5. Bacterial Growth Media

Lysogeny broth (LB) was used for most cases; only LBK was used for the positive assay, where NaCl was replaced with KCL at 10 gm/lt. All media contained ampicillin at 100 μg/mL.

## 3. Results

To evaluate the activity of both viroporins, we used bacteria-based assays in which the channel’s functionality changes the bacteria’s phenotype. The advantages of the assays are that they are amenable to high-throughput screening, and the ease of genetic manipulations in bacteria enables a rapid transition from one sequence/variant to another. Finally, these assays have been tested on numerous viroporins from various viruses [8,15,16,17,18,19,20].

### 3.1. Negative Assay

The first assay used to characterize both viroporins is one in which the protein is expressed at increasing levels in bacteria. As seen in other channels [8,15,16,17,18,19,20], elevated expression levels are detrimental to bacterial growth due to excessive permeabilization of the bacterial inner membrane. Therefore, in this assay, the viral protein eponymously impacts bacteria negatively.

**Figure 1:**
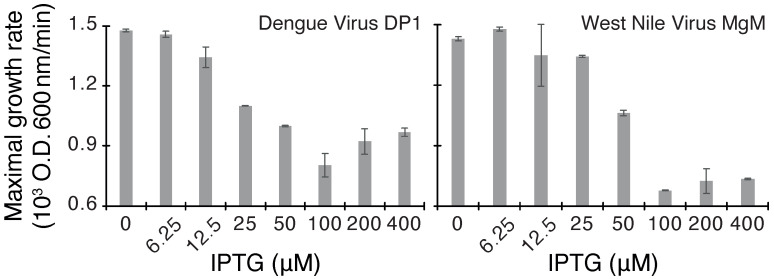
Negative assay of dengue virus DP1 and West Nile virus MgM viroporins. Maximal growth rates of DH10B bacteria expressing each viroporin are depicted as a function of the inducer concentration (IPTG).

As seen in Figure 1, both proteins are able to retard bacterial growth appreciably. At inducer concentrations of 50–100 μM, the growth rate is roughly half of what is observed without induction due to the negative influence of the channels on bacterial growth. Finally, we recognize that spurious factors can contribute to toxicity upon heterologous protein expression in bacteria. Therefore, we employed two additional bacteria-based assays to evaluate the channel activity of the viral proteins.

### 3.2. Positive Assay

The second bacteria-based assay we employed was reciprocal in nature to the negative assay listed above. $K^{+}$-uptake deficient bacteria are incapable of growing in regular bacteriological media unless it is enriched with potassium [21]. However, the bacteria are able to thrive in low $K^{+}$ media if they express a channel capable $K^{+}$ transport. Hence, in this instance, the channel’s activity impacts growth positively.

**Figure 2:**
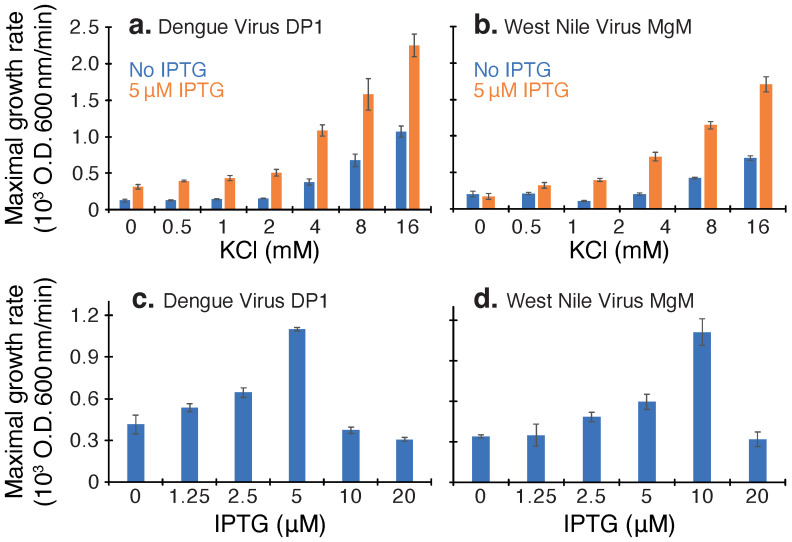
Positive assay of dengue virus DP1 and West Nile virus MgM viroporins. Panels (**a**,**b**): Maximal growth rates of $K^{+}$-uptake deficient bacteria expressing each viroporin monitored as a function of different $K^{+}$ concentrations. Growth enhancement can be observed upon comparing induced and uninduced bacteria in orange and blue, respectively. Panels (**c**,**d**): growth rates at different inducer concentrations (IPTG), as noted.

Results shown in Figure 2 indicate clearly that both viroporins are able to enhance the growth of $K^{+}$-uptake deficient bacteria. The experiments are repeated at several different $K^{+}$ concentrations to demonstrate the effect’s reproducible nature. Note that in this assay, the channel is expressed at a low level since higher levels will be once more detrimental to the bacteria [17], as seen in Figure 2c,d.

### 3.3. Acidity Assay

**Figure 3:**
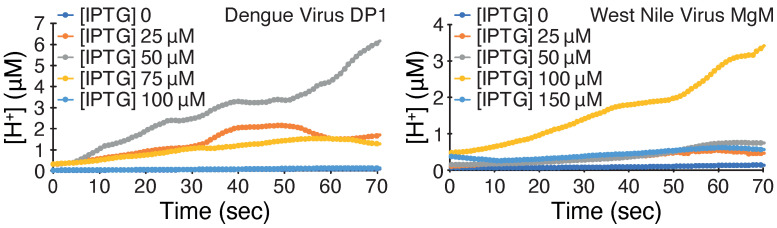
Acidity assay of dengue virus DP1 and West Nile virus MgM viroporins. Cytoplasmic $H^{+}$ concentration is monitored as a function time whereby at time 0, an acidic solution is injected into the media. Results are shown from bacteria in which viroporin induction is changed as a function of IPTG concentration as noted.

The final that we employed measured $H^{+}$ conductivity using bacteria expressing a pH-sensitive GFP [24]. This assay injects an acidic solution into the media while monitoring bacterial fluorescence indicative of its cytoplasmic pH. A change in fluorescence would indicate $H^{+}$ flux due to a channel conductivity [22]. The results shown in Figure 3 demonstrate that both viroporins can facilitate $H^{+}$ transport with dengue virus DP1 exhibiting higher activity than West Nile virus MgM.

### 3.4. Blocker Screening

Following the confirmation of channel activity of both viral proteins, we sought to identify drugs that could block their function. We employed the negative assay described above and screened a 2839 repurposed drug library. We subsequently followed by cross-checking every hit using the positive and pH-based assays. In the negative assay, a successful hit is expected to increase bacterial growth by abrogating the detrimental impact the viroporin has. In contrast, in the positive assay, successful blockers would impair bacterial growth since they would counteract the beneficial effect of the viral channel.

The reciprocal nature of both assays decreases the possibility of erroneous results. Specifically, compounds were classified as hits only if they increased bacterial growth in the negative assay while concomitantly reducing growth in the positive assay.

**Figure 4:**
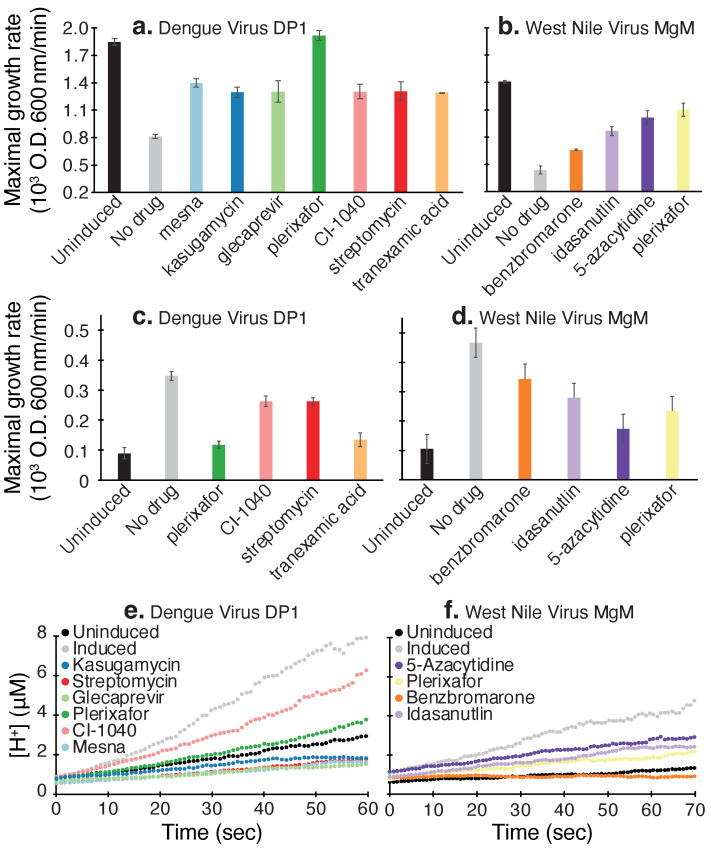
Blocker screening results against dengue virus DP1 and West Nile virus MgM viroporins. The negative assay results are depicted in panels (**a**,**b**), the results from the positive assay are presented in panels (**c**,**d**), and finally, the acidity assay results are presented in panels (**e**,**f**). Bacteria in which the channel expression was not induced or the drug was not added are used as controls. Blocker concentration was 50 μM.

The negative assay screen results are shown in Figure 4a,b, in which seven blockers were identified against dengue virus DP1 and four were identified against West Nile virus MgM. Subsequently, the positive assay was employed to examine the potency of the hits identified in the negative assay. Out of the seven blockers active against dengue virus DP1, only four were active in the positive assay (Figure 4c). In contrast, all hits in the negative assay against West Nile virus MgM were confirmed in the positive assay (Figure 4d).

Finally, the hits identified in the negative assay were examined in the acidity assay: Out of the seven blockers active against dengue virus DP1, six were confirmed in the acidity assay (Figure 4e). Once more, all hits from the negative and positive assay against West Nile virus MgM were active in the acidity assay (Figure 4f).

**Figure 5:**
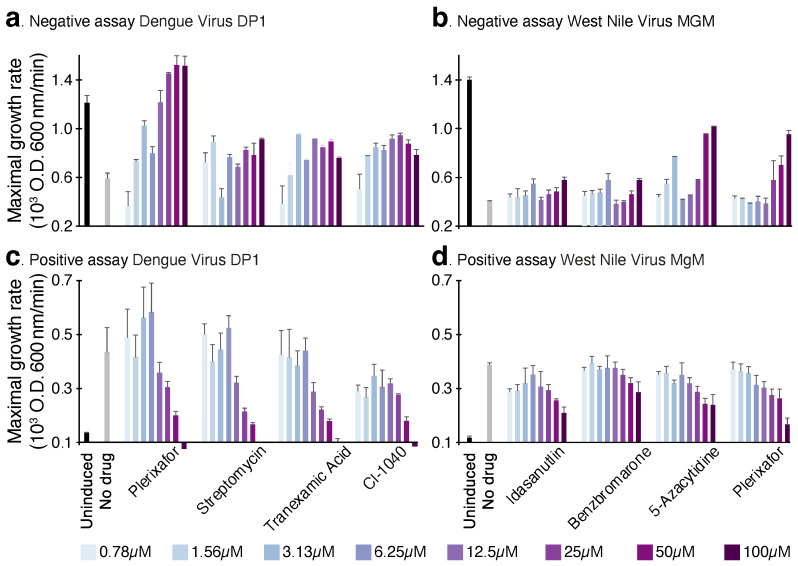
Blocker screening results against dengue virus DP1 and West Nile virus MgM viroporins. Maximal growth rates are depicted as a function of different compound concentrations (0.78–100 μM), as noted in the color legend. Bacteria in which the channel expression was not induced or the drug was not added are used as controls. Panels (**a**,**b**) present the negative assay results for dengue virus DP1 and West Nile virus MgM viroporins, respectively. Panels (**c**,**d**) present the positive assay results for dengue virus DP1 and West Nile virus MgM viroporins, respectively.

**Figure 6:**
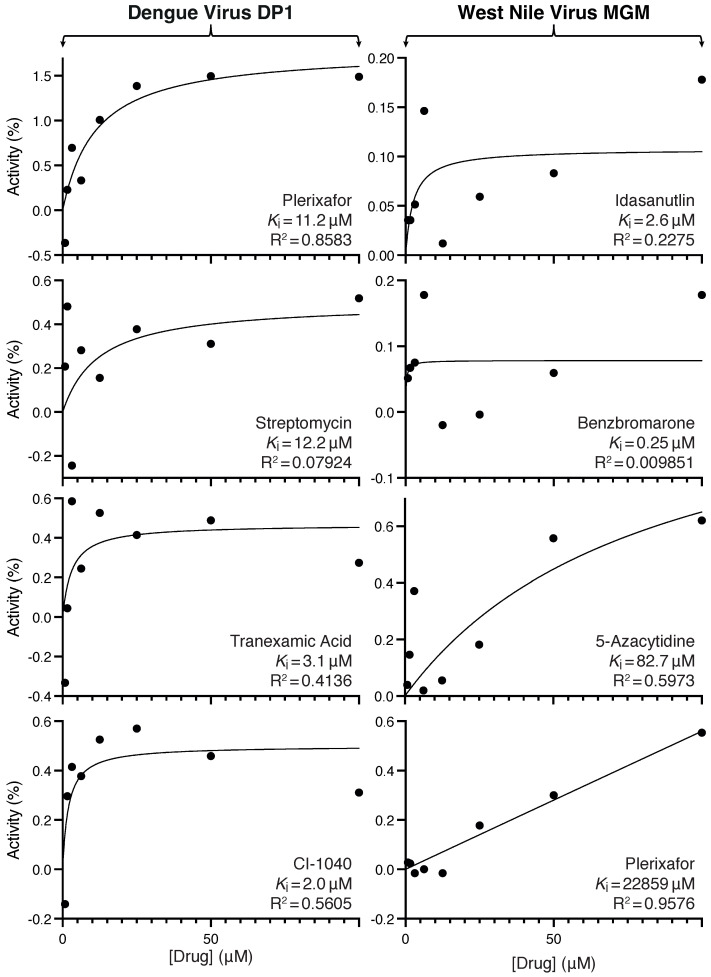
Inhibitory constants of blocker screening results against dengue virus DP1 (left column) and West Nile virus MgM (right column) viroporins using the negative assays (Figure 5a,b). Normalized growth enhancements are depicted as a function of different compound concentrations (0.78–100 μM). Bacteria in which the channel expression was not induced or the drug was not added are used as controls. The inhibitory constants are listed in each panel as well as the fitting coefficient.

The final step in the screening process involved dose–response analyses of the compounds that passed the negative and positive assays for each viroporin: plerixafor, streptomycin, tranexamic acid, and CI-1040 against dengue virus DP1, and idasanutlin, benzbro-marone, 5-azacytidine, and plerixafor against West Nile virus MgM. The results are depicted in Figure 5, and the fitted inhibitory contestants are provided in Figure 6.

### 3.5. Combination Testing

**Figure 7:**
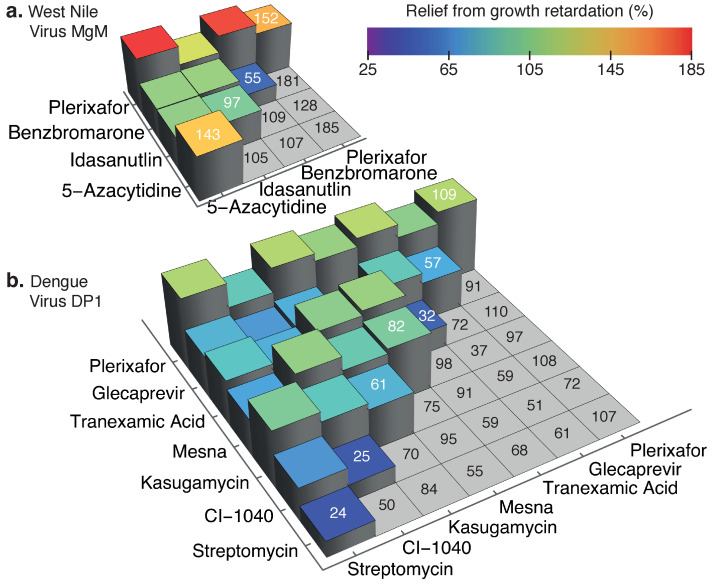
Blocker combinations against dengue virus DP1 and West Nile virus MgM viroporins. Results from the negative assay in which active blockers should improve growth rate relative to untreated samples. Different combinations are presented according to the color scale on the left and numerically on the right. Each drug was at 100 μM. Diagonal values in white indicate individual treatments.

We next sought to determine the potential additivity or synergism between the different hits. To that end, we examined all possible combinations of active drugs against dengue virus DP1 and West Nile virus MgM channels in equal concentration. Figure 7b shows that several dengue virus DP1 blocker combinations, such as tranexamic acid + mesna, kasugamycin + CI-1040, and kasugamycin + tranexamic acid exhibited additivity. Tranexamic acid + mesna, in particular, demonstrated 98% growth enhancement, whereas the impact of individual components was lower (32% and 82% for tranexamic acid and mesna, respectively). All the compounds combined with plerixafor exhibited better activity due to its masking effect. In the case of West Nile virus MgM (Figure 7a), combinations such as plerixafor + idasanutlin (128%) and plerixafor + benzbromarone (181%) exhibited additivity, while the effect of combining 5-azacytidine with idasanutlin (105%) reduced activity.

## 4. Discussion

This study aimed to identify inhibitors of proteins belonging to two flaviviruses as a means of curbing infectivity. We chose viroporins unique to each virus as drug targets since ion channels as a family are highly amenable to inhibition [25]. Subsequently, we employed bacteria-based assays to examine their activity and inhibition.

We started by establishing a baseline of protein activity in the three aforementioned assays. Both proteins were detrimental to bacterial growth when expressed at increasing levels (Figure 1), as expected from a channel that perforates the inner bacterial membrane [16]. In contrast, protein expression enabled $K^{+}$-uptake deficient bacteria to survive in low $K^{+}$ media (Figure 2), as expected from proteins that facilitate $K^{+}$ transport [21]. Finally, both proteins increased $H^{+}$ flux in bacteria (Figure 3), which is detected by a change in the fluorescence of a chromosomally encoded pH-sensitive GFP [22,24]. Hence, both proteins exhibited robust channel activity in all assays, similar to other viroporins [8,15,16,17,18,19,20].

In order to identify blockers, we screened a library of 2839 repurposed compounds, which includes FDA-approved drugs and those in advanced investigational stages. Drug repurposing has been proven a good option for new drug development, including antiviral research [26]. Many repurposed drugs have already been used as antiviral agents; for example, Digoxin and derivatives have been used to target HIV, HSV, and SARS-CoV-2 [27,28,29]. Recently, studies from our group have shown that repurposed drugs constitute a useful library from which to identify blockers of SARS-CoV-2 [8,18,19], which were later shown to possess antiviral activity in a tissue culture setting [30].

Our strategy employed positive and negative assays since their reciprocal behavior minimizes false positives. Hence, only compounds that score positively in both assays are considered by us a viable hit. Lastly, the acidity assay was used as a further independent check.

Starting with the negative assay, we identified seven blockers against dengue virus DP1 and four against West Nile virus MgM. Subsequently, we analyzed the hits from the negative assay in the positive and acidity-based assays: All compounds identified by the negative assay against West Nile virus MgM were confirmed by the other two assays. In contrast, out of the seven hits against dengue virus DP1 identified by the negative assay, only four were confirmed by the positive assay, and the acidity assay confirmed six.

Interestingly, the most efficacious blocker, plerixafor, inhibited both channels (Figure 5), perhaps due to the sequence similarity between the two proteins. At 50 μM, the compound increased the growth rate of bacteria by 150% when they express dengue virus DP1, as opposed to a more modest impact of 30% when they express West Nile virus MgM.

The differences between the efficacy of the drugs in the different assays may result from several factors: (i) The assays monitor similar albeit non-identical characteristics: prevention of membrane permeabilization versus blockage of $K^{+}$ or $H^{+}$ transport. (ii) The channel impact in the assays may not be linearly correlated. In conclusion, the assays validate each other qualitatively rather than quantitatively.

Upon testing the additive effects of drugs in both channels using the negative assay (Figure 7), we observed that plerixafor, in most cases, masks the effect of other drugs. However, tranexamic acid increases in promoting bacterial growth enhancement when used alongside kasugamycin and mesna.

In conclusion, our findings provide a list of blockers that inhibit dengue virus DP1 and West Nile virus MgM. Further studies employing appropriate bio-safety facilities are required to examine if the said compounds and their combinations exhibit antiviral activity in vitro and in vivo. Results of an identical approach used on SARS-CoV-2 have proven fruitful [30], indicating that channel blockers do indeed exhibit antiviral activity.

## Data Availability

Data is contained within the article.

## References

[1] GBD 2017 Causes of Death Collaborators (2018). Global, regional, and national age-sex-specific mortality for 282 causes of death in 195 countries and territories, 1980–2017: A systematic analysis for the Global Burden of Disease Study 2017. Lancet.

[2] Bhatt S., Gething P.W., Brady O.J., Messina J.P., Farlow A.W., Moyes C.L., Drake J.M., Brownstein J.S., Hoen A.G., Sankoh O. (2013). The global distribution and burden of dengue. Nature.

[3] Knipe D.M., Howley P.M. (2013). Fields Virology.

[4] Centers for Disease Control and Prevention (2020). West Nile Virus Disease Cases and Deaths Reported to CDC by Year and Clinical Presentation, 1999–2020.

[5] Redoni M., Yacoub S., Rivino L., Giacobbe D.R., Luzzati R., Di Bella S. (2020). Dengue: Status of current and under-development vaccines. Rev. Med. Virol..

[6] Pérez-Pérez M.J., Saiz J.C., Priego E.M., Martín-Acebes M.A. (2022). Antivirals against (Re)emerging Flaviviruses: Should We Target the Virus or the Host?. ACS Med. Chem. Lett..

[7] Rice C.M., Lenches E.M., Eddy S.R., Shin S.J., Sheets R.L., Strauss J.H. (1985). Nucleotide sequence of yellow fever virus: Implications for flavivirus gene expression and evolution. Science.

[8] Tomar P.P.S., Oren R., Krugliak M., Arkin I.T. (2019). Potential Viroporin Candidates From Pathogenic Viruses Using Bacteria-Based Bioassays. Viruses.

[9] Nieva J.L., Madan V., Carrasco L. (2012). Viroporins: Structure and biological functions. Nat. Rev. Microbiol..

[10] Gonzalez M.E., Carrasco L. (2003). Viroporins. FEBS Lett..

[11] Davies W.L., Grunert R.R., Haff R.F., Mcgahen J.W., Neumayer E.M., Paulshock M., Watts J.C., Wood T.R., Hermann E.C., Hoffmann C.E. (1964). Antiviral Activity of 1-Adamantanamine (Amantadine). Science.

[12] Hay A.J., Wolstenholme A.J., Skehel J.J., Smith M.H. (1985). The Molecular Basis of the Specific Anti-Influenza Action of Amantadine. EMBO J..

[13] Pinto L.H., Holsinger L.J., Lamb R.A. (1992). Influenza Virus M2 Protein Has Ion Channel Activity. Cell.

[14] Guan Y., Chen H. (2005). Resistance to anti-influenza agents. Lancet.

[15] Assa D., Alhadeff R., Krugliak M., Arkin I.T. (2016). Mapping the Resistance Potential of Influenza’s H+ Channel against an Antiviral Blocker. J. Mol. Biol..

[16] Astrahan P., Flitman-Tene R., Bennett E.R., Krugliak M., Gilon C., Arkin I.T. (2011). Quantitative analysis of influenza M2 channel blockers. Biochim. Biophys. Acta.

[17] Taube R., Alhadeff R., Assa D., Krugliak M., Arkin I.T. (2014). Bacteria-based analysis of HIV-1 Vpu channel activity. PLoS ONE.

[18] Tomar P.P.S., Krugliak M., Arkin I.T. (2021). Blockers of the SARS-CoV-2 3a Channel Identified by Targeted Drug Repurposing. Viruses.

[19] Tomar P.P.S., Krugliak M., Arkin I.T. (2021). Identification of SARS-CoV-2 E Channel Blockers from a Repurposed Drug Library. Pharmaceuticals.

[20] Tomar P.P.S., Krugliak M., Singh A., Arkin I.T. (2022). Zika M-A Potential Viroporin: Mutational Study and Drug Repurposing. Biomedicines.

[21] Stumpe S., Bakker E.P. (1997). Requirement of a Large K^+^-Uptake Capacity and of Extracytoplasmic Protease Activity for Protamine Resistance of *Escherichia coli*. Arch. Microbiol..

[22] Santner P., Martins J.M.d.S., Laursen J.S., Behrendt L., Riber L., Olsen C.A., Arkin I.T., Winther J.R., Willemoës M., Lindorff-Larsen K. (2018). A Robust Proton Flux (pHlux) Assay for Studying the Function and Inhibition of the Influenza A M2 Proton Channel. Biochemistry.

[23] McIlvaine T. (1921). A buffer solution for colorimetric comparison. J. Biol. Chem..

[24] Miesenböck G., De Angelis D.A., Rothman J.E. (1998). Visualizing secretion and synaptic transmission with pH-sensitive green fluorescent proteins. Nature.

[25] Scott C., Griffin S. (2015). Viroporins: Structure, function and potential as antiviral targets. J. Gen. Virol..

[26] García-Serradilla M., Risco C., Pacheco B. (2019). Drug repurposing for new, efficient, broad spectrum antivirals. Virus Res..

[27] Wong R.W., Balachandran A., Ostrowski M.A., Cochrane A. (2013). Digoxin suppresses HIV-1 replication by altering viral RNA processing. PLoS Pathog..

[28] Su C.T., Hsu J.T.A., Hsieh H.P., Lin P.H., Chen T.C., Kao C.L., Lee C.N., Chang S.Y. (2008). Anti-HSV activity of digitoxin and its possible mechanisms. Antivir. Res..

[29] Cho J., Lee Y.J., Kim J.H., Kim S.I., Kim S.S., Choi B.S., Choi J.H. (2020). Antiviral activity of digoxin and ouabain against SARS-CoV-2 infection and its implication for COVID-19. Sci. Rep..

[30] Singh A., Arkin I.T. (2022). Targeting Viral Ion Channels: A Promising Strategy to Curb SARS-CoV-2. Pharmaceuticals.

